# Influenza vaccination should have no border: cost-effectiveness of cross-border subsidy

**DOI:** 10.1186/s12889-021-11601-2

**Published:** 2021-08-12

**Authors:** Dan Yamin, Dor Kahana, Edan Shahmoon, Meagan C. Fitzpatrick, Alison P. Galvani

**Affiliations:** 1 Laboratory for Epidemic Modeling and Analysis, Department of Industrial Engineering, Faculty of Engineering, Tel Aviv University, 69978 Tel Aviv, Israel; 2 Center for Vaccine Development and Global Health, University of Maryland School of Medicine, Baltimore, 21201 Maryland USA; 3 Center for Infectious Disease Modeling and Analysis, Yale School of Public Health, Yale University, New Haven, CT 06510 USA

**Keywords:** Influenza vaccination, Transmission model, SIR model, Social contact, Cost-effective analysis

## Abstract

**Background:**

Influenza is a substantial cause of morbidity and mortality for Israel and the Palestinian territory. Given the extensive interaction between the two populations, vaccination in one population may indirectly benefit the other via reduced transmission. Due to the mobility and extensive contacts, Palestinians employed in Israel could be a prime target for vaccination.

**Methods:**

To evaluate the epidemiological and the economic benefits conferred by vaccinating Palestinians employed in Israel, we developed a model of influenza transmission within and between Israel and the West Bank. We parameterized the contact patterns underlying transmission by conducting a survey among Palestinians employed in Israel, and integrating survey results with traffic patterns and socio-demographic data.

**Results:**

Vaccinating 50% of Palestinian workers is predicted to reduce the annual influenza burden by 28,745 cases (95% CI: 15,031-50,717) and 37.7 deaths (95% CI: 19·9–65·5) for the Israeli population, and by 32,9900 cases (95% CI: 14,379-51,531) and 20.2 deaths (CI 95%: 9·8–31·5) for the Palestinian population. Further, we found that as the indirect protection was so substantial, funding such a vaccination campaign would be cost-saving from the Israeli Ministry of Health perspective.

**Conclusions:**

Offering influenza vaccination to Palestinians employed in Israel could efficiently reduce morbidity and mortality within both Israel and the Palestinian territory.

**Supplementary Information:**

The online version contains supplementary material available at 10.1186/s12889-021-11601-2.

## Background

Despite conflict between Israel and the Palestinian territories, economic, familial and cultural factors lead to extensive interaction between these two populations. Consequently, a high incidence of an infectious disease in one population can constitute a primary source of infection for the other [1]. While influenza is a persistent challenge across the globe, the disease is particularly problematic in the Middle East due to the exacerbation of transmission by demographic factors. Specifically, school-age children, who are disproportionately responsible for transmission [2, 3], represent 30 and 35% of Israeli and Palestinian populations, respectively, compared to 20% in the United States [4]. In addition, high population density increases the daily number of contacts [5]. In Israel alone, influenza is responsible for about 801,200 infections, 4130 hospitalizations and 1140 deaths annually [6]. This burden persists despite uptake that exceeds 60% within the targeted groups, and 20% overall. Influenza incidence in the Palestinian territories is unreliably reported but is likely to be even higher given the greater population density, [7] and the considerably low vaccination coverage [8–10].

Over 130,000 Palestinians from the West Bank are employed in Israel, corresponding to 4.3% of the population. These workers commute across the border on a daily basis. The majority are parents to young children, and thus at elevated risk of infection [11]. Additionally, about 12% of Israeli Arabs cross the border to the West Bank weekly for family visits and leisure time. These extensive contact patterns between Israelis and Palestinians, particularly within and between long-distance commuters in one of the world’s most densely populated regions, exacerbates transmission [12].

Individual’s infection risk is governed by their contacts [13]. Thus, prioritizing influenza vaccination for individuals with higher connectivity could curtail influenza transmission [14–16]. In practice, this includes the prioritization of school-aged children to reduce transmission in both children and adults [2, 3, 17]. Another form of reaching individuals with high contacts would be to prioritize those infected in previous seasons. This population has a disproportionate probability of being highly connected within contact networks and thus serve as the super-spreaders [18]. Here we show that vaccinating populations that may serve as a bridge for transmission between sub-groups with lower interactions, may be efficient to reduce transmission. Specifically, we quantify the extent to which extending influenza vaccination to Palestinians employed in Israel (PEI) has the potential to quell transmission on both sides of the border.

Cost-effectiveness analysis (CEA) of vaccination balances the vaccine program’s cost and the incremental health benefits attributable to the intervention. CEA typically accounts for the direct benefit of the vaccine in protecting the vaccinated individual. Here, we suggest a non-standard cost-effectiveness mechanism. We model the indirect health-economic benefit to one country generated from a vaccine’s subsidizing to a citizen from another country. Namely, the economic benefit is manifested by the indirect protection gained from reduced transmission (i.e. herd protection).

Specifically, while influenza vaccination is funded for the entire population in Israel, only vaccination of healthcare workers is subsidized by Palestinian authorities.

To evaluate the population-level impact of this targeted vaccination, we developed an influenza transmission model for Israel and the West Bank. The model incorporates socio-demographic data, weekly reported influenza and influenza-like-illness (ILI) diagnoses, influenza vaccination uptake among the Israeli population, and traffic volume data between Israel and the West Bank. Additionally, we conducted a survey to capture the social mixing patterns of PEI. We used this model to quantify the transmission within and between Israel and the Palestinian territory. We found that PEI are at elevated risk of becoming infected. This sub-population also served as a bridge for transmission between the two territories. We, therefore, evaluated the effectiveness and cost-effectiveness of extending Israeli-subsidized vaccination to these Palestinians. We found that this strategy would substantially reduce influenza morbidity and mortality on both sides of the border. Even based solely on the societal benefit to Israel, it would be very cost-effective for Israel to fund vaccination for these Palestinian workers.

## Methods

The study was approved by Tel Aviv University’s institutional review board, signed by Prof. Eran Dolev, on March 1, 2017.

**Fig. 1:**
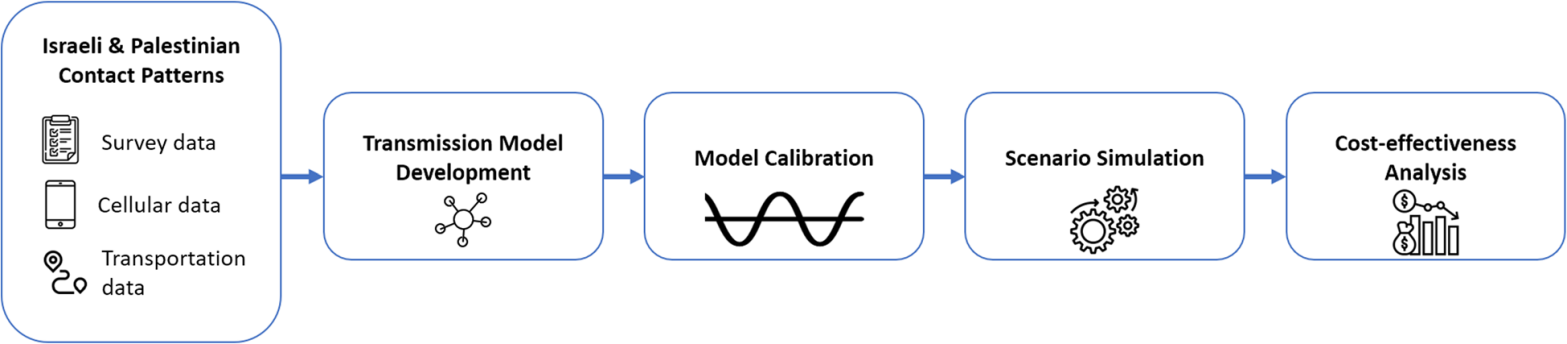
Study scheme flowchart

The overall methodological framework of the study from chronologic order is presented in Fig. 1. Briefly, we developed a model of influenza transmission within and between Israel and the West Bank. We parameterized the contact patterns underlying transmission by conducting a survey among Palestinians employed in Israel. We integrated the survey results with traffic patterns and socio-demographic data. Next, we calibrated the epidemiological parameters of our transmission model with influenza records. We then simulated the transmission model under a wide variety of vaccination coverage levels to evaluate the effectiveness and cost-effectiveness of influenza vaccination for Palestinians employed in Israel.

### Transmission model overview

**Fig. 2:**
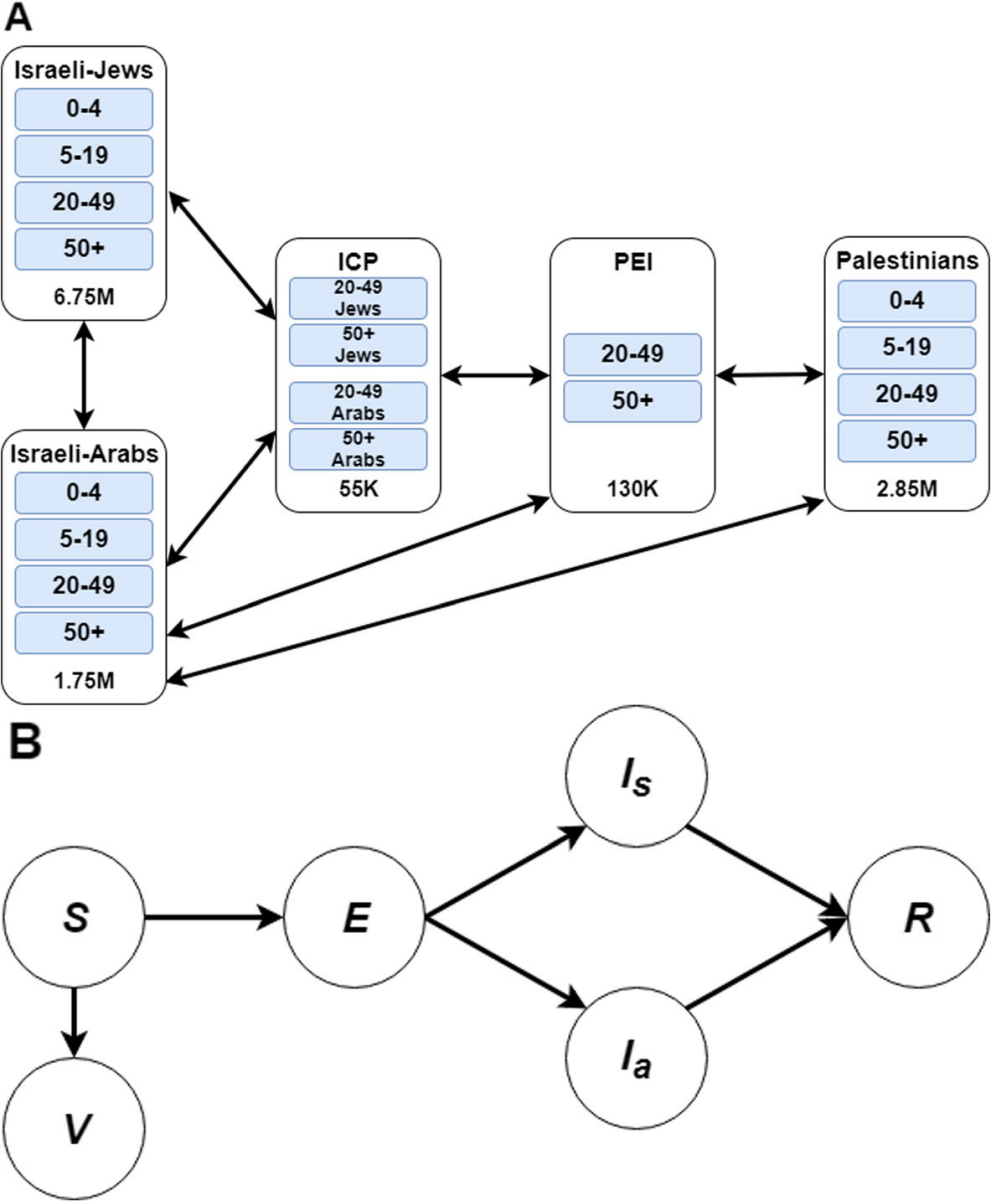
Structure of the model. (**A**) Diagram of the contact mixing patterns between and within the Israeli and the Palestinian populations. Each component represents a disjoint group and specifies its size and age stratification. The connections between components describe contacts between individuals from each group. The groups Palestinians employed in Israel (PEI) and Israelis in close contact with PEI (ICP) interact directly with each other and serve as a bridge between the general Israeli and Palestinian populations. There is also direct contact between the Israeli-Arabs and the Palestinian groups due to family visits. (**B**) Compartmental diagram of the transmission model. Susceptible individuals start in compartment S, from which they may become infected but not yet infectious and transition to the compartment E, until they move to an infectious compartment. Infectious individuals can be either symptomatic I_s_ or asymptomatic I_a_, until recovery, when they move to compartment R. Individuals who are effectively vaccinated transition to the V compartment, and are fully protected for the entire season

We developed a dynamic model for influenza transmission within and between Israel and the Palestinian territory (Fig. 2A). Our compartmental framework [19] stratifies the population into health-related compartments. Transitions between the compartments are governed by a series of difference equations (Fig. 2B). We further stratified the population into five groups based on their ethnicity and employment: Israeli Jews, Israeli Arabs, Palestinians employed in Israel (PEI), Israelis in contact with Palestinians (ICP) and the rest of the Palestinian population (Fig. 2A and Additional file 1, p 3–7). The ICP group includes individuals in direct contact with PEI as part of their workday, such as soldiers at checkpoints, bus drivers, and Israeli colleagues. Additionally, each group is age-stratified to account for demographic differences in epidemiological parameters and mixing patterns (Fig. 2A). Altogether, our model included 18 subgroups based on the specification of age, ethnicity, and employment.

At the beginning of each season, susceptible individuals from subgroup *j* are in the *Sj* compartment, from which they may become infected and transition to the exposed compartment *Ej*. Following an incubation period, exposed individuals move to an infectious compartment, either symptomatic *l*_*j*_^*S*^ or asymptomatic *l*_*j*_^*A*^. Upon recovery, individuals transition to the *Rj* compartment. Due to cross-reactive antibodies elicited by previous exposures, we consider an age-specific fraction of each subgroup to be immune at the beginning of each influenza season. This fraction of individuals remain in the immune/recovered compartment for the duration of the influenza season (Additional file 1, pp. 4). As influenza vaccine efficacy is imperfect, we considered only a proportion of vaccinated individuals to be protected against infection [20]. Susceptible individuals for whom vaccine was effective transition to the *Vj* compartment. Individuals in both *Rj* and *Vj* are protected for the season. Parameter values are given in the Additional file 1, pp. 10–14.

#### Force of infection

The rate at which an individual from subgroup *i* infects an individual from subgroup *j* depends on a combination of three factors: 1) subgroup-specific susceptibility for *j*, 2) subgroup-specific contact rates, and 3) seasonality in the force of infection. Combining these components, the daily force of infection *λ*_*j*_(*t*) for an individual in subgroup *j* is given by:

$$ {\lambda}_j(t)={\beta}_j\cdot {\sum}_{k\in \left\{s,a\right\}}\kern1.5em {\sum}_{i\in N}\kern1.5em {C}_{i,j}\cdot {l_i}^k\kern1.5em \left(t-1\right)\cdot \left(1+\cos \left(\frac{2\pi t}{365}+\phi \right)\right) $$

where *β*_*j*_ is the probability of infection for an individual from subgroup *j,* given an infectious contact from an individual and was calibrated to explicitly consider the variation in the risk of infection for different subgroups based on their age and geographical location (see model calibration). *C*_*i*, *j*_ denotes the contact mixing rates between infected host *i* and susceptible individual *j*.⋅*l*_*i*_^*k*^ represents the number of infectious individuals with category *k*, depending on whether the infection is symptomatic or asymptomatic. Seasonality is incorporated via the function $\left(1+\mathit{\cos}\ \left(\frac{2\pi t}{365}+\phi \right)\ \right)$, previously shown to accurately capture the seasonal variations of influenza incidence in Israel [21].

### Contact mixing patterns

We explicitly accounted for the different mixing patterns between the 18 subgroups modeled (Fig. 2A). The contact mixing patterns between these subpopulations has not previously been described. Therefore, we estimated these rates by combining published age-specific contact diaries for European countries [22] with region-specific data, including a prospective survey that we conducted at border checkpoints. Additional data sources included Israeli locational data derived from cell phone records and daily traffic volume data between Israel and the West Bank.

Age-dependent daily mixing patterns were derived from a previous survey that was conducted in eight European countries and was adjusted to 152 additional countries, including Israel and Jordan [5]. We applied the Israeli-specific matrix for the mixing rates between Israelis. As there is no Palestinian-specific matrix, we applied the Jordanian matrix for mixing within the Palestinian territory. Jordan borders the West Bank and shares socio-demographic and cultural similarities with the Palestinian territory.

To account for the difference in contact patterns between Israeli Arabs and Israeli Jews, we derived a contact matrix from a recent analysis of the movements of 200,000 cell phone users over 2 months combined with ethnicity data from the Israeli Central Bureau of Statistics [23]. These data reveal that Israeli Arabs and Israeli Jews have fewer contacts with each other, compared to mixing within their respective groups (Additional file 1, pp. 5–6). To evaluate the contact mixing patterns of the PEI subpopulation, we conducted in-person surveys between March 2, 2016, and May 4, 2016 (Table S1 Additional file 1, detailed survey data are available Additional file 2: In-person survey raw data). This time frame corresponds to the end of the 2016 influenza season. The survey was administered in Arabic to 500 PEI between the ages of 19–62 at seven cross-border checkpoints. The survey included general socio-demographic questions as well as detailed questions regarding daily contacts. These contact data revealed frequent mixing between similar age groups, moderate mixing between children and people of their parents’ age, and infrequent mixing among other age groups. In addition, extensive contact was revealed between the PEI and ICP groups.

Israeli Arabs and the Palestinian population interact most often during the weekends for family visits and leisure time. To quantify these contacts, we used traffic volume data from the Israeli Central Bureau of Statistics (CBS). Weekend traffic volumes on roads crossing from Israel to the Palestinian cities in the West Bank were used to estimate contacts between the two subpopulations.

### Vaccination coverage

We extracted the vaccination coverage for the Israeli population from the electronic medical records of Maccabi health maintenance organization (HMO). Maccabi HMO covers 25% of the Israeli population, providing a substantial sample of the country’s population. These electronic medical records (EMR) include longitudinal data of 250,000 members (randomly assigned), between 2013 and 2017. Each patient is affiliated with one of 138 clinics of Maccabi [23, 24]. In addition, demographic data is available, including age. We used this data to estimate the vaccination coverage for each of the subgroups considered in the model in each of the five influenza seasons.

For the PEI, vaccination coverage was determined, based on the survey, was 4.4%. As for the general Palestinian population, data is unreliably reported. Nevertheless, it has been recently shown that even among healthcare workers, vaccination coverage in the west bank was only 21% [25]. We conservatively considered data from Jordan, a neighboring country with similar demographics and economic characteristics to the Palestinian territories, indicating that the typical vaccination coverage is 2% [26].

### Model parameterization and calibration

When possible, distributions for epidemiological parameters were derived from published literature (Additional file 1, pp. 7–8). We calibrated the seasonal offset, *ϕ*, as well as the age and country specific probabilities of infection given an infectious contact, *β*_*j*_. We calibrated these parameters to weekly reported influenza cases for Israel. To incorporate region-specific seasonality, we extracted age-stratified weekly influenza and influenza-like-illness cases occurring between 2013 and 2017 from the electronic medical records of Maccabi health maintenance organization (HMO). To account for underreporting and misdiagnosis, we scaled the number of cases such that the mean annual incidence for Israel would range between 20 and 30% in children and 5–10% in adults, consistent both with WHO estimates [27] and with a recent meta-analysis of randomized controlled trials [7]. For the base case, we then calibrated the age-specific transmission parameters to correspond to midpoints of 15 and 7.5% for children and adults, respectively. The full ranges were considered in the uncertainty analyses, as detailed below.

For calibration, we used the Nelder-Mead simplex algorithm [28] to select values for the empirically uncertain parameters which maximized the likelihood of the incidence data from five seasons (2013–2017). Furthermore, we assessed the balance between including additional parameters and the danger of overfitting the model using the Akaike information criterion (AIC) [29], a measure of model fitting derived from information theory. Specifically, we used the AIC to determine the number of age classes that would optimally be parameterized by the available data (Additional file 1, pp. 8–10).

### Calculation of secondary cases

To quantify the contribution of each group to influenza transmission, we calculated the average number of secondary cases generated per infected individual in a given subgroup [30]. Throughout the influenza season that spans from September 1 to April 30, we calculated the daily rate at which an individual within subgroup *i*, would transmit to individuals from subgroup *j* (Additional file 1, pp. 15). The summation of these daily rates across all age strata in a group is equivalent to the total number of new cases for which members of the group had been the source of infection.

### Predictive simulations

To determine the population-level impact of a policy targeting the PEI group for influenza vaccination, we simulated five influenza seasons. For each of the five seasons simulated, we considered vaccination uptake of 25, 50 and 75% for the PEI population as well as no of vaccination uptake. We then evaluated the annual number of influenza cases averted with the PEI vaccination program. Namely, we calculated the yearly difference in the total number of infected cases with and without vaccination for each vaccination uptake scenarios.

### Cost-effectiveness analyses

The epidemiological results generated by the model simulations were integrated with an economic evaluation to estimate the cost-effectiveness of influenza vaccination for PEI. For the economic evaluation, we considered the direct costs of four mutually exclusive clinical outcomes from the perspective of the Israeli government: 1) home care, 2) outpatient visit, 3) hospitalization, or 4) death (Table S2). Notably, with the exception of over the counter medications, no out-of-pocket expenses are required for outpatient visits or hospitalizations associated with influenza and its complications. The probability of each outcome is age-dependent [6]. We combined these outcome probabilities with model predictions to estimate the number of clinical outcomes averted for Israel and the Palestinian territory. Vaccination costs included the listed vaccine price of $15 per dose and an administration cost of $1.25 per person, and are fully covered by the government [6]. To account for the impact of influenza on quality of life, we included an outcome-specific loss of quality-adjusted-life-years (QALYs) (Additional files 1, 14). The number of QALYs saved per death averted was calculated using the age of death and the life expectancy in Israel. Inline with Israel economic growth rate in recent years, costs and QALYs were discounted at 3.5%.

We applied criteria suggested by the WHO that defines interventions as cost-effective when the cost of saving a life-year is lower than three times the annual per capita gross domestic product (GDP). Interventions are considered very cost-effective when this cost is lower than the per capita GDP [31]. We specifically evaluated the cost-effectiveness of subsidizing an influenza vaccination campaign with 50% uptake among PEI, from the perspective of the State of Israel.

### Uncertainty analysis

We also conducted a probabilistic global uncertainty analysis to integrate the uncertainty in epidemiological and cost parameters. Specifically, we sampled 1000 coupled sets of incidence rates for Israel and the Palestinian territory. Annual incidence was sampled from ranges spanning 5–10% for adults and 20–30% for children, which is consistent with WHO estimates. Due to the younger population in the Palestinian territory, overall annual incidence differed between Israel and the Palestinian territory even though identical age-specific incidence was applied to the two regions. Specifically, overall incidence ranged between 9.8–16.4% in Israel, and between 11.4–18.6% in the Palestinian territory. We then independently calibrated the other transmission model parameters to meet the sampled incidence value for each of the 1000 simulations. These epidemiological variations arises from 1) uncertainty regarding background immunity arising from exposure in previous seasons (i.e. the initial proportion of susceptible individuals), and 2) uncertainty regarding the probability of infection given contact between susceptible and infectious persons. To account simultaneously for these two components, we randomly sampled plausible levels of age-specific background immunity, and calibrated model parameters to generate the yearly incidence rates sampled.

Costs were sampled from normal distributions with the 95% confidence interval set to values 20% above and below the base case. We conducted 10,000 simulations by independently sampling an epidemiological scenario and a set of monetary values. We then used this probabilistic analysis to evaluate the sensitivity of our conclusion across variation in willingness-to-pay for QALYs. At each value for willingness-to-pay, we calculated the proportion of simulations in which vaccination of PEI has an incremental cost-effectiveness ratio below that threshold. This proportion corresponds to the probability that vaccination of PEI would be cost-effective, and was calculated independently for three levels of coverage (25, 50, 75%).

## Results

To characterize the demography, infection rate, and contact patterns of Palestinians employed in Israel, we conducted in-person surveys between March 2, 2016, and May 4, 2016, which corresponds to the end of the 2016 influenza season. Our survey included 500 participants at seven cross-border checkpoints, aged 19–62 (IQR: 26–36), of whom 35% reported influenza-like symptoms during the 2016 season. The median household size was 5 (IQR 4–6) individuals, of which 16.2% (IQR: 0–25·0%) included children < 5 y, and 33% (IQC 0–60·0%) included children between 5 and 18 y. Participants reported 67% (95% IQR: 10–126%) more daily contacts than the average for an Israeli of the same age [5], and 9.1% (3.8–16.7%) of their contacts were with Israelis. These interactions occurred across multiple locations, including public transportation, the checkpoint, and the workplace in Israel. In short, this subpopulation consists of larger household size and more extensive and more widespread contacts.

**Fig. 3:**
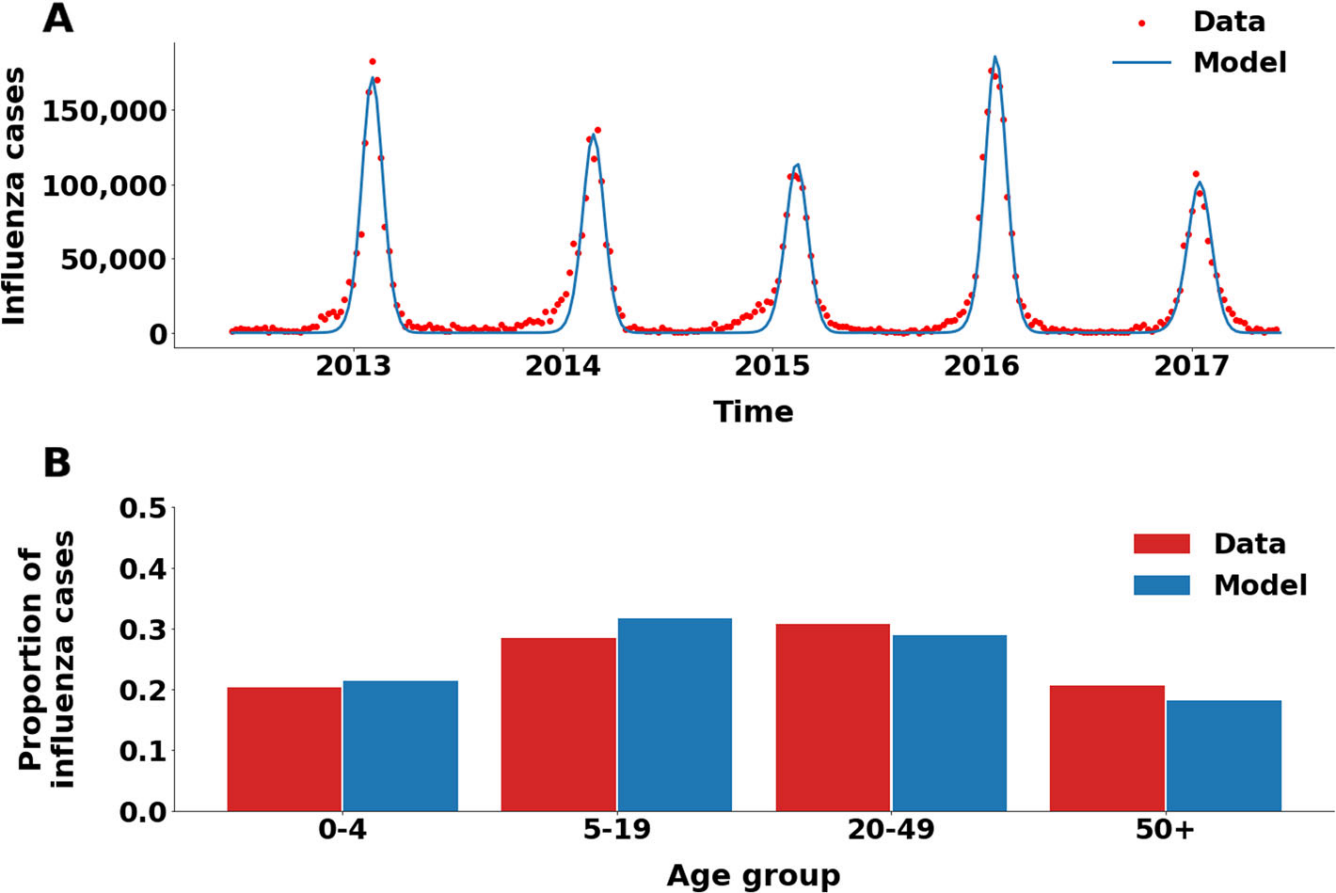
(**A**) Time series of recorded weekly influenza cases in Israel and model fit for 2013–2017 seasons. (**B**) Age distribution of influenza cases in Israel, according to the model (blue) and the data (red)

We integrated these empirical results into a model of influenza transmission within and between Israel and the Palestinian territory. The transmission model included 18 subgroups to account for socio-demographic mixing patterns in Israel and in the West Bank (Fig. 2A). The model was calibrated to age-stratified influenza and ILI weekly reported cases for the Israeli population across five seasons (2013–2017), and to the annual estimates of influenza incidence in each age group for the Palestinian population. Our model replicated the data with a correlation of 85% (Fig. 3A, B). Thus, the calibrated model accurately replicated the epidemiological trajectories for all five seasons, as well as the considerable variability between seasons.

**Fig. 4:**
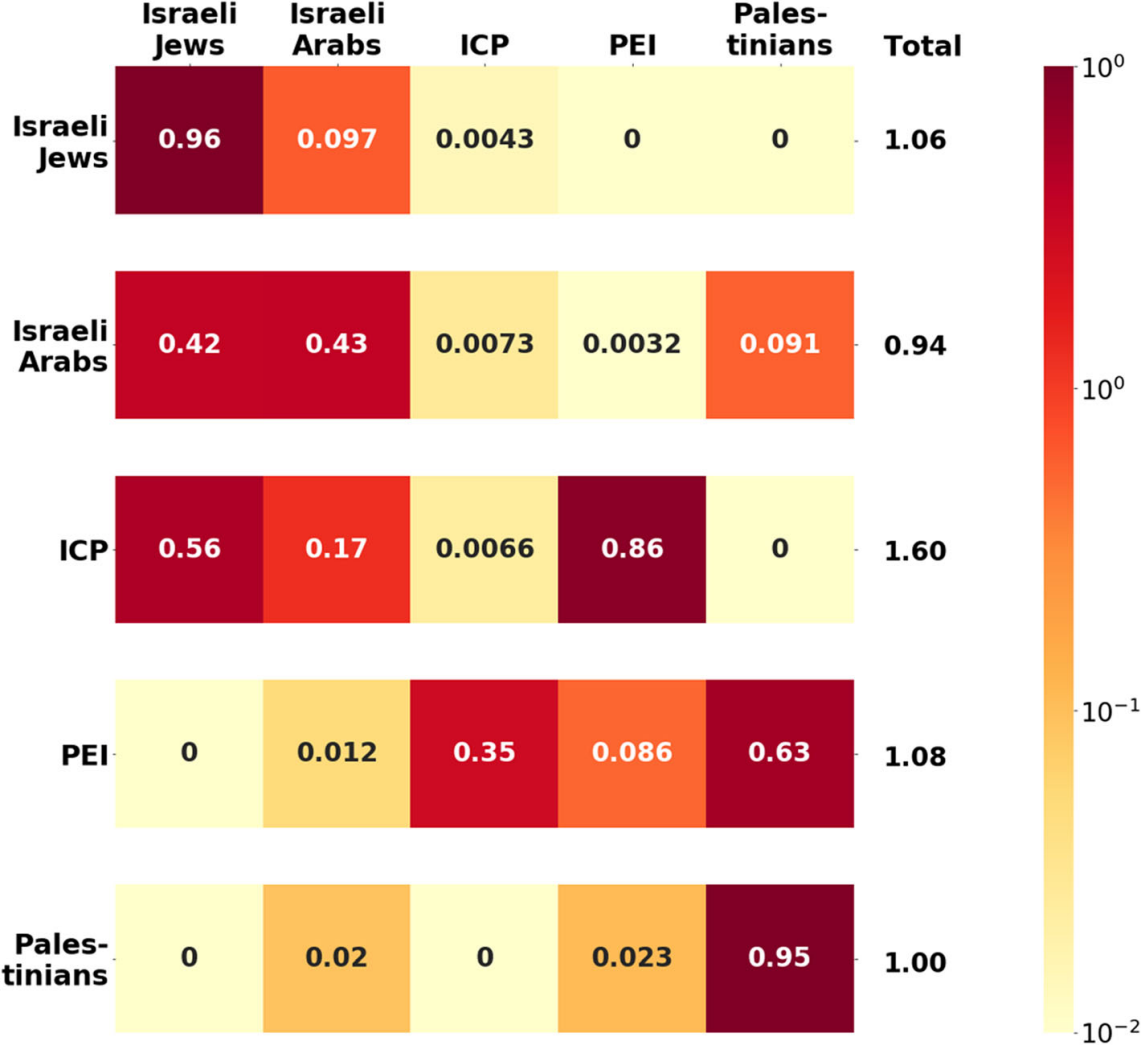
Average number of secondary infections generated per case for each group. A row indicates the source of infection, and a column indicates the group becoming infected. The color and number at the intersection of the row and column indicate the average number of secondary infections expected from a single infective case

While the majority of transmission occurs within each country, we found that PEI and ICP infect each other with higher rates than any other sub-population (Fig. 4). Specifically, the average infected PEI will infect 1.08 individuals in total, 32.5% from the ICP subgroup (Fig. 4). Likewise, the average infected ICP is expected to infect 1.60 individuals, 53.8% from the PEI. Consequently, our findings underscore that the PEI and the ICP serve as bridges between Israel and the Palestinian territory. This finding was robust to sensitivity analyses (Fig. 4).

We found both that Palestinians interact with more children than the Israelis and that children are substantially more likely to transmit influenza than any other age group. An infected child under 4 years of age would transmit influenza to 1.23 individuals, while an infected adult > 50 would transmit, on average, to 0.51 individuals (Additional file 1, p 11). Frequent interactions with children contribute to the relatively high incidence in PEI.

**Fig. 5:**
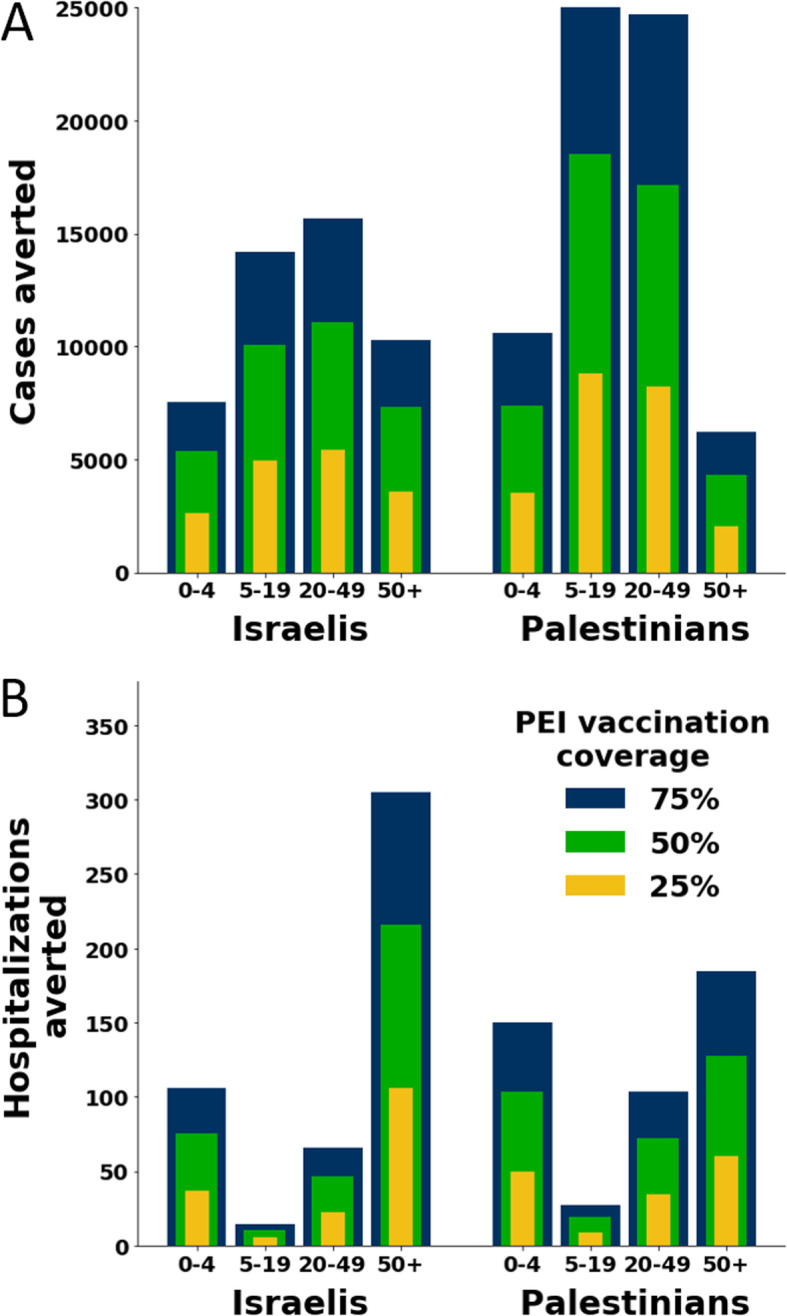
Model predictions of influenza cases averted (**A**) and hospitalizations averted (**B**) for Israelis and Palestinians for each age group, assuming vaccination coverage of 25, 50 and 75% of the PEI group. These vaccination coverages correspond to 32,500, 65,000, and 97,500 influenza vaccination doses, respectively

Using our transmission model, we evaluated the population-level impact of influenza vaccination within the PEI group. Specifically, we analyzed the number of influenza cases and hospitalizations averted (Fig. 5 A and B), in Israel and Palestinian territory across a range of vaccination uptake. We found that a substantial reduction in influenza infection is anticipated not only for PEI, but also for the other groups. For example, with 50% uptake among PEI, every 10 vaccine doses are predicted to avert 3·0 cases in Israel and 6·4 cases in the Palestinian territory. Most of the averted cases would be among individuals between the ages of 5–49 years, while the majority of the averted hospitalizations would be among individuals under 4 years and older than 50 years, as these age groups have the highest risk of influenza complications (Fig. 5). Accordingly, influenza vaccination within the PEI group is anticipated to substantially reduce transmission and severe outcomes to both Israelis and Palestinians.

We performed a cost-effectiveness analysis from the perspective of the Israeli government. We estimated the cost of vaccination as well as the medical costs averted in 2018 USD. Health was measured in quality-adjusted life years (QALYs), a metric that combines morbidity and mortality. We found that the programmatic cost for vaccinating 50% of PEI would be $1,186,250. This campaign would save 567 (95% CI: 301–991) QALYs and avert $1,700,400 (CI: $910,170-2,979,500) of medical costs for Israel, compared to the status quo. This expenditure would therefore be cost-saving from the perspective of the Israeli Ministry of Health. Additional benefits include 396 (CI: 186–618) QALYs saved for the Palestinians. Campaigns remain cost-saving across vaccination campaign coverage examined ranging from 25 to 75%. Thus, in any realistic coverage setting, funding the suggested policy is beneficial from the perspective of the Israeli government.

**Fig. 6:**
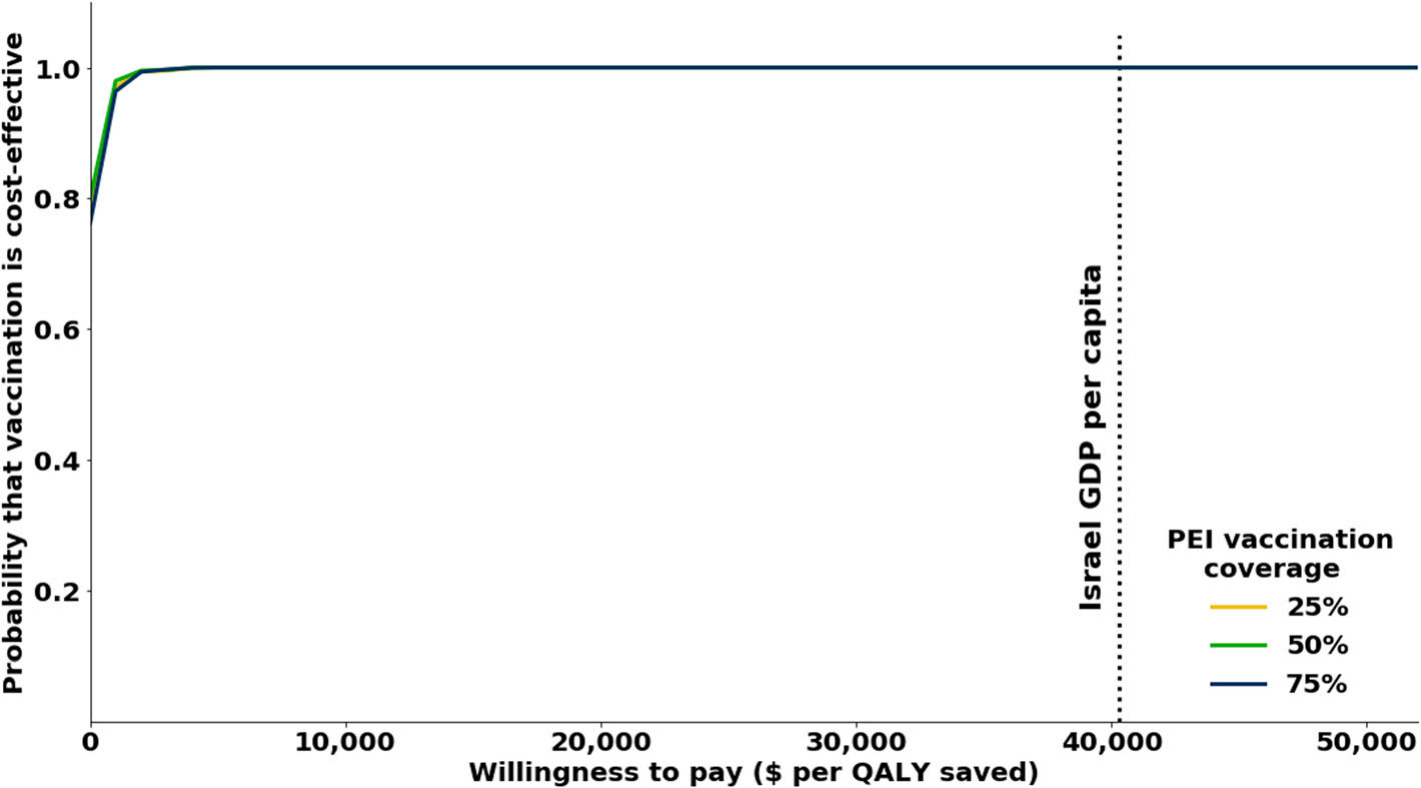
Probabilities that fully subsidizing vaccination for PEI would be cost-effective, across different levels of willingness to pay for QALYs. This policy is predicted to be cost-saving with probability 0·8 and very cost-effective with probability 0·98, according to WHO criteria

We conducted a global uncertainty analysis which integrated variation from the epidemiological, monetary, and vaccine-related parameters. Our global uncertainty analysis determined that vaccinating 50% of Palestinian workers is predicted to reduce the annual influenza burden by 28,745 cases (95% CI: 15,031-50,717) and 37.7 deaths (95% CI: 19·9–65.5) for the Israeli population, and by 32,9900 cases (95% CI: 14,379-51,531) and 20.2 deaths (CI 95%: 9.8–31.5) for the Palestinian population. Furthermore, we found that vaccination of PEI is 80% likely to be cost-saving, such that the averted medical costs associated with GP consultations and hospitalizations in Israel exceeds the cost of fully subsidizing vaccination for the PEI group. We also found that vaccination of PEI is 98% likely to be very cost-effective according to WHO criteria (Fig. 4). In all 10,000 simulations tested in the global uncertainty analysis, we found that the suggested policy would be cost-effective (Fig. 6). Results were not sensitive to vaccination coverage levels within the range considered. Namely, the marginal contribution per vaccinated was not affected by the vaccination coverage, demonstrating the importance of subsidizing as many individuals as possible.

## Discussion

Our results indicate that influenza vaccination of Palestinians employed in Israel could substantially reduce morbidity and mortality within both Israel and the Palestinian territory. PEI are a relatively small group of about 130,000 individuals, equivalent to 4.5% of the Palestinian population, and to 1.5% of the Israeli population. Nevertheless, they play an instrumental role in transmission. Even when we solely considered the benefit to Israel, our analysis predicted that influenza vaccination for PEI would be very cost-effective.

These results arise primarily from the unique contact patterns of the PEI over a wide spatial range. Specifically, PEI commute long distances spanning all across Israel. Furthermore, PEI have daily contact with a relatively large number of both Israelis and Palestinians from different populations and age groups, including soldiers at the checkpoints, colleagues at work and in public transportation. By contrast, interaction between the general Israeli and Palestinian populations is rare, and thus the PEI serve as a bridge between the two.

Beyond the PEI, we identified several populations that also disproportionately contribute to transmission. For example, ICP were found to have even more secondary cases per infected individual than PEI. However, as vaccination is already subsidized for Israelis, we did not consider inclusion of this population in the PEI-focused campaign. Additional interventions should be implemented to improve vaccine uptake in this critical group. For example, uptake increases substantially when vaccines are offered in the workplace or at other accessible locations [32, 33]. Given that a large proportion of ICP are employed at or near the checkpoints, campaigns to vaccinate individuals at these locations regardless of nationality would be a highly effective approach to promote coverage in both bridge groups. More generally, future studies can model the effectiveness of the targeting of other groups that may serve as bridge groups such as cross-border students, and health workers.

Significant losses in productivity are attributable to influenza. Thus, economic analyses generally find vaccination to be cost-effective for society [34]. This efficiency primarily arises from direct protection of the vaccinated employee. Here, we conservatively showed that even when ignoring the losses in productivity and considering only the direct costs, influenza vaccination of Palestinians employed in Israel is economic. Moreover, in this unique analysis, the economic benefit arises solely from indirect protection, as we did not account in the cost-effectiveness analysis the benefit of preventing disease for the Palestinians. Regardless, we found that the reduction in infection among the recipient’s contacts is substantial enough to justify the vaccination costs. This finding suggests that economic analyses which do not consider the indirect effects of influenza vaccination may be substantially underestimating their actual value.

The rapid growth of the SARS-CoV-2 pandemic led to unprecedented control measures on a global scale. These measures severely impacted more impoverished regions with low technological abilities, and population in needs. Given the similar transmission patterns between the SARS-CoV 2 and influenza [35], vaccinating the PEI against COVID-19 is likely to be highly effective to reduce the disease burden and save lives in both countries.

As for any modeling study, we made a number of simplifying assumptions. There is currently no reliable data regarding influenza incidence in the West Bank. To account for this uncertainty, we conducted sensitivity analyses across a wide range of annual influenza rates, corresponding to observations from other developed and developing countries. The finding that vaccination of PEI would be cost-effective is robust across the wide range of epidemiological parameters assessed in our sensitivity analyses.

## Conclusions

Offering influenza vaccination to Palestinians employed in Israel could efficiently reduce morbidity and mortality within both Israel and the Palestinian territory. Cooperation in healthcare between neighboring countries, despite conflict, can provide economic and humanitarian benefits to both sides [1, 36]. A mutual campaign to increase influenza vaccination uptake among Palestinians employed in Israel can reduce morbidity, severe infection and mortality for both populations. As a practical approach, vaccinations should be subsidized by Israel and offered on a voluntary basis to those waiting at the checkpoints, potentially by a third party such as the United Nations Relief and Rehabilitation Administration (UNRRA). Our findings underscore that in the fight against infectious diseases, and in particular against influenza, vaccination should have no border.

## Supplementary Information

**Additional file 1.** Appendix for: Influenza vaccination should have no border: cost-effectiveness of cross-border subsidy.

**Additional file 2.** In-person survey raw data.

## Data Availability

All data that support the findings of this study are publically available. The in-person survey raw data is presented in this study as ‘Additional file 2’.

## Abbreviations

CBS: Central Bureau of Statistics
HMO: Health maintenance organization
ICP: Israelis in contact with Palestinians that are employed in Israel
ILI: Influenza-like illness
PEI: Palestinians employed in Israel
QALY: Quality-adjusted life year

## References

[1] Hermesh B, Rosenthal A, Davidovitch N (2019). Rethinking “one health” through brucellosis: ethics, boundaries and politics. Monash Bioeth Rev.

[2] Galvani AP, Reluga TC, Chapman GB (2007). Long-standing influenza vaccination policy is in accord with individual self-interest but not with the utilitarian optimum. Proc Natl Acad Sci U S A.

[3] Yamin D, Gavious A (2013). Incentives’ Effect in Influenza Vaccination Policy.

[4] World Population Prospects - Population Division - United Nations. https://population.un.org/wpp/Download/Standard/Population/. Accessed 9 June 2020.

[5] Prem K, Cook AR, Jit M (2017). Projecting social contact matrices in 152 countries using contact surveys and demographic data. PLoS Comput Biol.

[6] Yamin D, Balicer RD, Galvani AP (2014). Cost-effectiveness of influenza vaccination in prior pneumonia patients in Israel. Vaccine.

[7] Somes MP, Turner RM, Dwyer LJ, Newall AT (2018). Estimating the annual attack rate of seasonal influenza among unvaccinated individuals: a systematic review and meta-analysis. Vaccine.

[8] Abusrewil S, Algeer A, Aljifri A, Al Slail F, Andrew MK, Awad Tag Eldin M (2019). Influenza surveillance in Middle East, north, east and South Africa: report of the 8th <scp>MENA</scp> influenza stakeholders network. Influenza Other Respir Viruses.

[9] Abubakar A, Melhem N, Malik M, Dbaibo G, Khan WM, Zaraket H (2019). Seasonal influenza vaccination policies in the eastern Mediterranean region: current status and the way forward. Vaccine.

[10] Nazzal Z, Dmaidi L, Hamshari Y. Influenza Vaccine Uptake among Palestinian Hospitals’ Health Care Workers: Barriers and Motivators. Jacobs J Community Med. 2015;1(2):013.

[11] Medlock J, Galvani AP (2009). Optimizing influenza vaccine distribution. Science.

[12] Charaudeau S, Pakdaman K, Boëlle PY (2014). Commuter mobility and the spread of infectious diseases: application to influenza in France. PLoS One.

[13] Bansal S, Pourbohloul B, Meyers LA (2006). A comparative analysis of influenza vaccination programs. PLoS Med.

[14] Dezső Z, Barabási AL (2002). Halting viruses in scale-free networks. Phys Rev E - Stat Physics, Plasmas, Fluids, Relat Interdiscip Top.

[15] Pastor-Satorras R, Vespignani A (2002). Immunization of complex networks. Phys Rev E - Stat Physics, Plasmas, Fluids, Relat Interdiscip Top.

[16] Woolhouse MEJ, Dye C, Etard JF, Smith T, Charlwood JD, Garnett GP, Hagan P, Hii JLK, Ndhlovu PD, Quinnell RJ, Watts CH, Chandiwana SK, Anderson RM (1997). Heterogeneities in the transmission of infectious agents: implications for the design of control programs. Proc Natl Acad Sci U S A.

[17] Longini IM, Halloran ME, Nizam A, Yang Y (2004). Containing pandemic influenza with antiviral agents. Am J Epidemiol.

[18] Yamin D, Gavious A, Solnik E, Davidovitch N, Balicer RD, Galvani AP, Pliskin JS (2014). An innovative influenza vaccination policy: targeting last Season’s patients. PLoS Comput Biol.

[19] Vynnycky E, White R (2010). An introduction to infectious disease modelling.

[20] Osterholm MT, Kelley NS, Sommer A, Belongia EA (2012). Efficacy and effectiveness of influenza vaccines: a systematic review and meta-analysis. Lancet Infect Dis.

[21] Bock Axelsen J, Yaari R, Grenfell BT, Stone L (2014). Multiannual forecasting of seasonal influenza dynamics reveals climatic and evolutionary drivers. Proc Natl Acad Sci U S A.

[22] Mossong JL, Hens N, Jit M, Beutels P, Auranen K, Mikolajczyk R, Massari M, Salmaso S, Tomba GS, Wallinga J, Heijne J, Sadkowska-Todys M, Rosinska M, Edmunds WJ (2008). Social contacts and mixing patterns relevant to the spread of infectious diseases. PLoS Med.

[23] Kahana D, Yamin D (2021). Accounting for the spread of vaccination behavior to optimize influenza vaccination programs. PLoS One.

[24] Shaham A, Chodick G, Shalev V, Yamin D (2020). Personal and social patterns predict influenza vaccination decision. BMC Public Health.

[25] Nazzal Z, Dmaidi L, Hamshari Y (2015). Influenza Vaccine Uptake among Palestinian Hospitals’ Health Care Workers: Barriers and Motivators.

[26] Palache A, Oriol-Mathieu V, Fino M, Xydia-Charmanta M (2015). Seasonal influenza vaccine dose distribution in 195 countries (2004-2013): Little progress in estimated global vaccination coverage. Vaccine.

[27] WHO/Europe | Influenza - Data and statistics. https://www.euro.who.int/en/health-topics/communicable-diseases/influenza/data-and-statistics. Accessed 9 June 2020.

[28] Gao F, Han L (2012). Implementing the Nelder-Mead simplex algorithm with adaptive parameters. Comput Optim Appl.

[29] Burnham KP, Anderson DR, Burnham KP. Model selection and multimodel inference : a practical information-theoretic approach: Springer; 2002.

[30] Yamin D, Jones FK, JP DV, Gertler S, Kobiler O, Townsend JP (2016). Vaccination strategies against respiratory syncytial virus. Proc Natl Acad Sci U S A.

[31] Edejer T, Baltussen R, Adam T, Hutubessy R, Acharya A, Evens D (2002). WHO guide to cost-effectiveness analysis.

[32] Yamin D, Gavious A, Davidovitch N, Pliskin JS (2014). Role of intervention programs to increase influenza vaccination in Israel. Isr J Health Policy Res.

[33] Shahrabani S, Benzion U (2010). Workplace vaccination and other factors impacting influenza vaccination decision among employees in Israel. Int J Environ Res Public Health.

[34] Ting EEK, Sander B, Ungar WJ (2017). Systematic review of the cost-effectiveness of influenza immunization programs. Vaccine.

[35] Yechezkel M, Weiss A, Rejwan I, Shahmoon E, Ben Gal S, Yamin D. Human mobility and poverty as key drivers of COVID-19 transmission and control. medRxiv. 2020:2020.06.04.20112417. 10.1101/2020.06.04.20112417. 10.1186/s12889-021-10561-x PMC7993906 33765977

[36] Gottlieb N, Filc D, Davidovitch N (2012). Medical humanitarianism, human rights and political advocacy: the case of the Israeli open clinic. Soc Sci Med.

