# Supplementary material for: Influenza vaccination should have no border: cost-effectiveness of cross-border subsidy
Source: BMC Public Health. 2021 Aug 12;21:1543. doi: 10.1186/s12889-021-11601-2 (PMC8358262; doi:10.1186/s12889-021-11601-2)
Supplement: Supplementary file 1 — Additional file 1. Appendix for: Influenza vaccination should have no border: cost-effectiveness of cross-border subsidy. [file 12889_2021_11601_MOESM1_ESM.doc]

Appendix for: Influenza vaccination should have no border: cost-effectiveness of cross-border subsidy

Dan Yamin (PhD)^1*^ , Dor Kahana (M.Sc.) ^1*^, Edan Shahmoon (B.Sc.)^1^, Meagan C. Fitzpatrick (PhD)^2,3^, Alison P. Galvani (PhD)^3#^

^1^Department of Industrial Engineering, Faculty of Engineering, Tel Aviv University, Tel Aviv 69978, Israel

^2^ Center for Vaccine Development and Global Health, University of Maryland School of Medicine, Baltimore, MD 21201
^3^ Center for Infectious Disease Modeling and Analysis, Yale School of Public Health, Yale University, New Haven, CT 06510
* Contributed equally
# To who correspondence should be addressed: email: alison.galvani@gmail.com , +1(203)785-2642

Contents

[**1.** **The transmission model** 2](#__RefHeading___Toc78796207)

[*Model transitioning* 3](#__RefHeading___Toc78796208)

[*Force of infection* 4](#__RefHeading___Toc78796209)

[**2.** **Data set and parameters** 5](#__RefHeading___Toc78796210)

[*Checkpoint survey* 5](#__RefHeading___Toc78796211)

[*Population structure* 7](#__RefHeading___Toc78796212)

[*Contact mixing patterns* 8](#__RefHeading___Toc78796213)

[**3. Fixed parameters** 10](#__RefHeading___Toc78796214)

[*Cross reactivity* 10](#__RefHeading___Toc78796215)

[**4. Calibrated parameters** 11](#__RefHeading___Toc78796216)

[**5. Cost-effectiveness Analysis** 14](#__RefHeading___Toc78796217)

[**6. Additional results** 15](#__RefHeading___Toc78796218)

# **The transmission model**

We developed a dynamic model for influenza transmission within and between Israel and the Palestinian territory (figure 1A). Our model is a modified Susceptible-Exposed-Vaccinated-Infected-Recovered (SEVIR) compartmental framework, whereby the population is stratified into health-related compartments, and transitions between the compartments occur over time. To explicitly account for age-specific and occupation-specific variation in contact-mixing patterns, we further stratified the population into 18 subgroups (detailed below). Altogether, we stratified the population into 6×18=108 compartments based on their subgroup *j* and one of the following health related states: susceptible
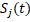
, vaccinated
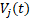
, exposed
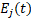
, symptomatic infectious
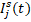
, asymptomatic infectious,
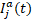
, and recovered
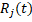
, such that at any given time *t* (in days):

| 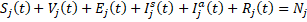, | (1) |
| --- | --- |

where the index
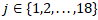
 specifies the subgroups of each individual.
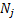
 is the population size for each subgroup, and is assumed, for simplicity, to be fixed.

## *Model transitioning*

At the beginning of each season, susceptible individuals from subgroup *j* are in the
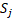
 compartment, from which they may become infected and transition to the exposed compartment
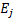
. Following an incubation period, exposed individuals move to an infectious compartment, either symptomatic
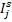
 or asymptomatic
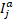
. Upon recovery, individuals transition to the
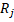
compartment. Due to cross-reactive antibodies elicited by previous exposures, we consider an age-specific fraction of each subgroup to be immune at the beginning of each influenza season. As influenza vaccine efficacy is imperfect, we considered only a proportion
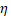
of vaccinated individuals to be protected against infection.[1] Susceptible individuals for whom vaccine was effective transition to the
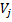
 compartment. Individuals in both
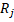
 and
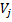
, including those with protection arising from influenza exposure in previous seasons[2], are fully protected for the entire season. Thus, the transmission model is composed of the following system of difference equations:

| 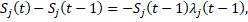  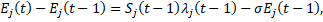  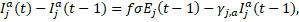  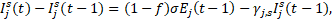  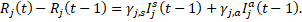 | (2) |
| --- | --- |

Accounting for vaccination at the beginning of an influenza season, the initial conditions are set to:

| 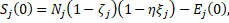  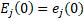,  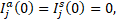  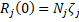,  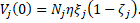 | (3) |
| --- | --- |

At the beginning of each season, individuals will belong to the vaccinated compartment
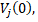
 if a) they were vaccinated, whereby
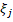
 is the fraction of vaccinated individuals, and b) the vaccine was effective
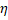
, and c) they were not already protected due to cross-reactive antibodies elicited by previous exposures
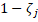
. By the same logic, we calculated
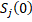
 and
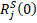
.
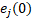
 represents the initial number of exposed individuals and corresponds to the seed of the system.

## *Force of infection*

The rate at which an individual from subgroup *i* infects an individual from subgroup *j* depends on a combination of three factors: 1) subgroup-specific susceptibility, 2) subgroup-specific contact rates, and 3) seasonality in the force of infection. Combining these components, the force of infection
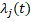
 for an individual in subgroup *j* is given by:

| 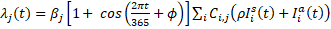, | (4) |
| --- | --- |

where
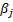
 is the susceptibility rate for an individual from subgroup *j,* given an infectious contact
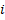
.
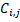
 denotes the contact rate between infected host
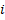
 and susceptible individual
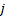
. We included a relative infectivity parameter
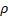
, to account for the observation that individuals symptomatically infected have, on average, higher infectious viral load than those asymptomatically infected. Seasonality is incorporated via the function
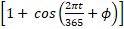
, previously shown to accurately capture the seasonal variations of influenza incidence.[3]

# **Data set and parameters**

## *Checkpoint survey*

To characterize the demography and contact patterns within the group of Palestinians employed in Israel (PEI), we conducted an in-person survey at the Israeli side of seven cross-border checkpoints: Tarkomia, Maale Edomim, Barta, Naalin, Kalandia, Beit Lechem, and Hader. The survey, authorized by an institutional review board headed by Prof. Eran Dolev, was approved on March 1, 2017. The survey was executed by do-et[4] , a survey company that has over 13 years of experience working with immigrants and minorities. The survey was conducted between March 2 and May 4, 2017, corresponding to the end of the 2016-2017 influenza season. All participants read and signed the consent form before participating in the study.

The survey was administered in Arabic to 500 PEI between the ages of 19-62 (Table S1). A do-et representative observed the survey to ensure all details and questions were clear. The survey included general sociodemographic questions, questions related to influenza-illness and vaccination status, as well as detailed questions regarding daily contacts.

Sociodemographic questions included gender, age, type of work in Israel and household size. Additionally, participants were asked to report their type of work in Israel, and the number of days per week worked in Israel. Health-related questions included whether or not the participant was vaccinated against influenza this season, the location at which they were vaccinated and the reason for their decision. Additionally, participants were asked if they thought they had experienced influenza illness this year.

Questions regarding contacts were formulated in a similar framework as previous studies[5, 6]. Specifically, contacts included skin-to-skin contact such as a kiss or handshake, and two-way conversations of at least three words in the physical presence of another person. Participants were asked to report the age group (0-4, 5-19, 20-49, 50 and above), ethnicity (Israeli-Jew, Israeli-Arab, Palestinian), and the number of unique contacts they experienced within the past 24 hours, and to categorize the contact as a) close to their home town, b) during leisure and pray time, c) during travel to and from work within Israel, d) at the checkpoint, e) during their travel to and from work within the Palestinian territory, and f) at work and during lunch break.

**Table S1: In-person survey questions**

|  | Question Item | | | | | |
| --- | --- | --- | --- | --- | --- | --- |
| **Sociodemographic Items** | 1. What is your gender? | | | | | |
|  | 1. What is your age (in years)? | | | | | |
|  | 1. How many people live in your household, including yourself? | | | | | |
|  | 1. What is your current primary occupation? | | | | | |
|  | 1. Try to recall all the days on which you entered Israel last week (circle the days):   Sunday/ Monday/ Tuesday/ Wednesday/ Thursday/ Friday/ Saturday | | | | | |
| **Influenza-related Items** | 1. Did you receive a flu shot during the recent influenza season? (Yes/ No/ I don’t remember)   Please answer sections 1A and 1B if you answered YES:  1A. Where did you receive a flu shot? ____  1B. What was the main reason(s) for your decision? ____ | | | | | |
|  | 1. Try to recall whether you experienced an influenza-like illness during the recent influenza season? (Yes/ No/ I don’t remember) | | | | | |
| **Contacts** | For the following questions, we define a **‘contact**’ as an encounter that includes a conversation with more than three words or any form of physical contact, including a kiss or handshake. | | | | | |
|  | Try to estimate the number of contacts you had at your home or neighborhood during the 24 hours of yesterday | | | | | |
|  | # <5 years old | # 5-17 years old | | # 18-50 years old | | # >50 years old |
|  | Try to estimate the number of contacts you had on your way to or from work during prayer or leisure time, **excluding the contacts you already reported** **on**, during the 24 hours of yesterday | | | | | |
|  | # <5 years old | # 5-17 years old | | # 18-50 years old | | # >50 years old |
|  | Try to estimate the number of contacts you had at work, including meal breaks, **excluding the contacts you already reported on**, during the 24 hours of yesterday | | | | | |
|  | # <5 years old | # 5-17 years old | | # 18-50 years old | | # >50 years old |
|  | Overall, try to estimate how many people you were in contact with during the 24 hours of yesterday | | | | | |
|  | # Jews (who are residents of Israel) | | # Arabs who are residents of Israel | | # Arabs who are residents of the Palestinian authority | |

## *Population structure*

For the model to explicitly account for the varying contact mixing pattern as well as demographic differences in epidemiological parameters, we stratified the population into five groups based on their ethnicity and type of employment: Israeli Jews, Israeli Arabs, Israelis in contact with Palestinians (ICP), Palestinians employed in Israel, and the rest of the Palestinian population. The ICP group included individuals in direct contact with PEI as part of their workday.

According to the Palestinian Central Bureau of Statistics, 130,000 Palestinians are employed in Israel, 92% of which are between the ages 19-49 and 8% are over 50. The vast majority of them live in the west bank. These workers commute across the border on a daily basis during the weekdays. The ICP includes 1) soldiers at checkpoints and Police officers at borders, 2) colleagues at work, and 3) Israelis that encounter Palestinians on the bus to work sites in Israel. There are approximately 80 active checkpoints between Israel and the West Bank. We estimated ~20 Israeli Jewish soldiers at each checkpoint per day, which corresponds to 1,600 soldiers. The number of co-workers and their ethnicity were estimated from the survey and revealed a ratio of one Israeli to 3.6 PEI. Conservatively to our findings, we assumed that the only contacts on the bus are with the transit driver, corresponding to one Israeli driver per eight PEI. Altogether, the ICP group included 55,000 individuals. The size of the other three groups was derived from the Israeli Central Bureau of Statistics (CBS) and subtraction of the sizes of the PEI and ICP.

## *Contact mixing patterns*

We explicitly accounted for the different mixing patterns between the 18 subgroups modeled (figure S1). The contact mixing patterns between these subpopulations have not previously been described. Therefore, we estimated these rates by combining published age-specific contact diaries for European countries[5] with region-specific data, including the survey that was conducted at border checkpoints. Additional data sources included longitudinal data derived from cell phone records and data on daily traffic volume between Israel and the West Bank.

Age-dependent daily mixing patterns were derived from a previous survey that was conducted in eight European countries and was adjusted to 152 additional countries, including Israel and Jordan.[7] We applied the Israeli-specific matrix for the mixing rates between Israelis. As there is no Palestinian-specific matrix, we applied the Jordanian matrix for mixing within the Palestinian territory. Jordan borders the West Bank and shares sociodemographic and cultural similarities to the Palestinian territory.

To account for the difference in contact patterns between Israeli Arabs and Israeli Jews, we derived a contact matrix from a recent analysis of the movements of 1,800,000 cell phone users over two months combined with ethnicity data from the CBS.[8] These data reveal that Israeli Arabs and Israeli Jews have fewer contacts with each other, compared to mixing within their respective groups.

To evaluate the contact mixing patterns of the PEI subpopulation, we analyzed the in-person survey. These contact data revealed frequent mixing between similar age groups, moderate mixing between children and people of their parents’ age, and infrequent mixing among other age groups. In addition, extensive contact was revealed between the PEI and ICP groups. The number of contacts was multiplied by 5/7 as all participants reported working in Israel on weekdays.

Israeli-Arabs tend to visit their families and spend their leisure time in the West Bank on weekends. In order to account for these family visits and to estimate the number of Israeli-Arabs visiting the West Bank every Saturday, we used traffic volume data from the CBS. The data includes traffic volume (number of vehicles) at each road section for each day of the week. We assumed all traffic on the road sections crossing from Israel to the West Bank on Saturdays as Israeli-Arab visitors, as the vast majority of the Israeli-Jewish population living in the West Bank area are religious and do not drive on Saturdays (Shabat). The total number of visitors was the number of vehicles according to the traffic volume data multiplied by four individuals per vehicle. The number of visitors was divided by seven as these contacts occur only once a week (on Saturdays).

To generate the contact matrix used in our model and ensure the matrix is symmetric, we adjusted the contact matrix according to the means for reciprocal subgroup pairings, following Medlock and Galvani[9]. The final resulted adjusted contact matrix is displayed in figure S1.


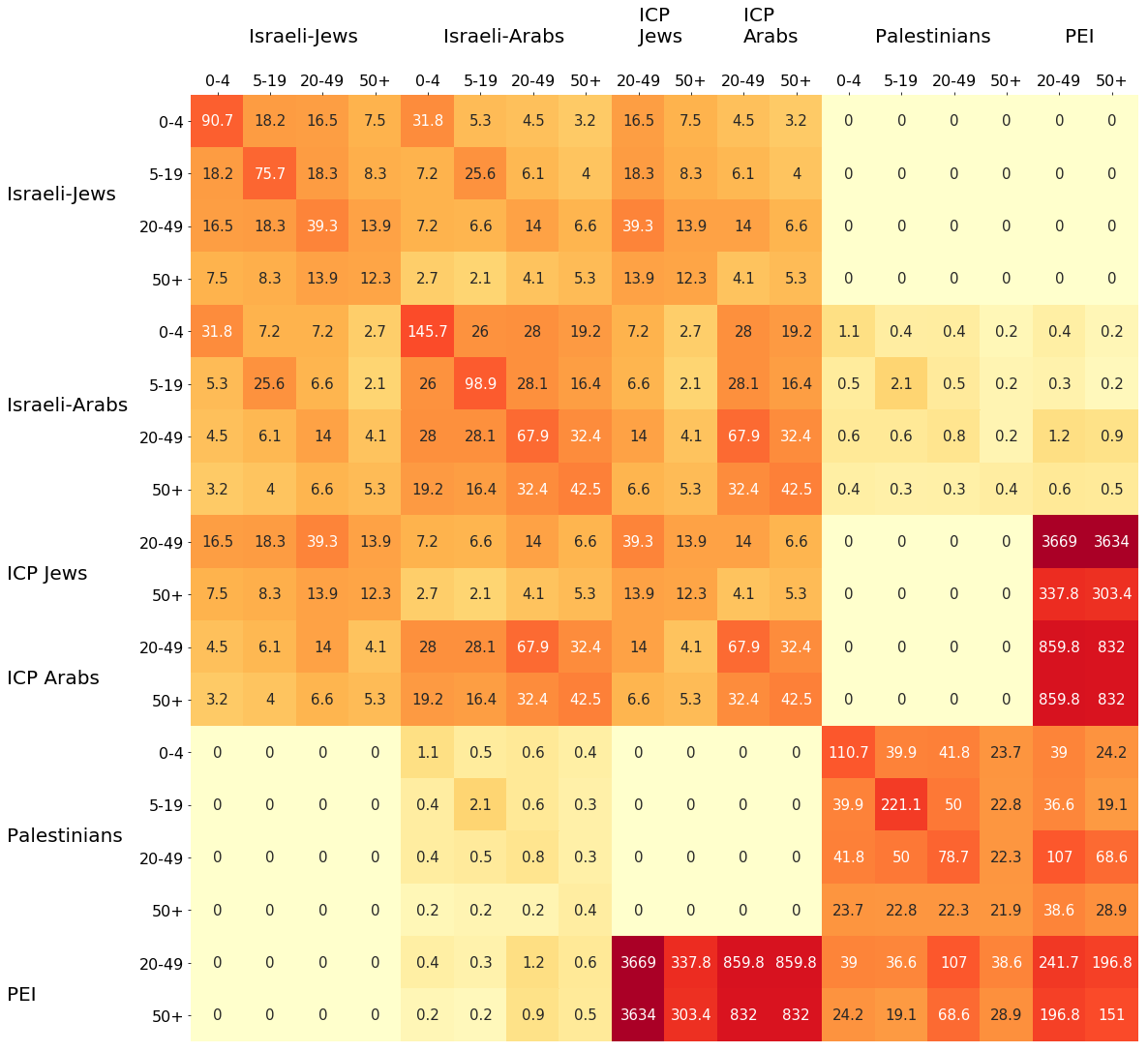


**Figure S1:** **Adjusted contact mixing pattern.** Contact mixing patterns between the 18 subgroups of the model. The matrix is adjusted for the contact matrix according to the means for reciprocal subgroup pairings

# **3. Fixed parameters**

The model’s fixed parameters are shown in Table S2.

## *Cross reactivity*

Cross-reactive antibodies play an important role in understanding and correctly modeling the transmission of influenza, as it affects the portion of the population which is susceptible to the disease. The cross-reactivity rates differ with age and vary dramatically between seasons. To deal with these changes and in order to take into account the uncertainty they cause, we checked a broad range of cross-reactivity rates for each age group, based on different studies from Israel, the US, and Germany (Table S2). These model settings were checked in the global uncertainty analysis we have performed.

**Table S2: epidemiological parameters considered in the transmission model and their justification from the relevant literature**

| **Parameter** | **Description** | **Value** | **Justification** |
| --- | --- | --- | --- |
| 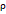 | Relative infectivity of symptomatic infection versus asymptomatic infection | 1.22 | [10] |
| 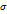 | 1/incubation period | 0.5 | [11] |
| 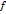 | Probability to become asymptomatic given infection. | 0.191 | [12, 13] [14] |
| 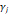 | 1 / recovery rate of an infected (symptomatic / asymptomatic  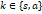) individual in age group j. | For k=s:  0-4: 1/7  5-19: 1/7  20+: 1/7  For k=a: 1/3.2 | [15, 16] |
| 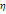 | Influenza vaccination efficacy | 0.45 (29-60) | [17] |
| 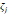 | Probability to be protected against vaccination due to exposure in previous seasons. | 0-4: 0 (0, 0.30)  5-19: 0.2 (0.036, 0.60)  20-49: 0.3 (0.16, 0.7)  50+: 0.25 (0.17, 0.7) | [2, 18–21] |

## **4. Calibrated parameters**

The model includes several missing parameters: the seasonal offset,
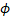
, as well as subgroup-specific probabilities of infection given an infectious contact,
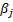
. To estimate these empirically unknown epidemiological parameters, we calibrated our model to weekly reported influenza cases in Israel. To incorporate region-specific seasonality, we extracted age-stratified weekly influenza and influenza-like-illness cases occurring between 2013 and 2017 from the electronic medical records of Maccabi HMO. To account for underreporting and misdiagnosis, we scaled the number of cases such that the mean annual incidence for Israel would range between 20-30% in children and 5-10% in adults, consistent with both WHO estimates[22] and a recent meta-analysis of randomized controlled trials[23]. For the base case, we then calibrated the age-specific transmission parameters to correspond to midpoints of 15% and 7.5% for children and adults, respectively. The full ranges were considered in the uncertainty analyses.

To calibrate the model to the incidence data we minimized the squared error between model predictions and incidence data. This is equivalent to maximum likelihood estimation assuming a normal distribution of the error. We used the Nelder-Mead simplex algorithm [24] to select values for the empirically uncertain parameters which maximized the likelihood of the incidence data from five seasons (2013-2017). Our calibrated model included five parameters for each season: the seasonal offset,
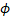
, subgroup-specific probabilities of infection given an infectious contact,
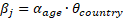
 such that
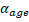
 was aggregated to age groups
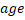
:0-4, 5-49, and 50 and above, and
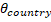
 was set to one for Israelis and estimated for the Palestinians. This way, we could maintain a low number of parameters with a high correlation fit.

To determine whether we could further reduce the number of free parameters, we evaluated two other model structures, both with four parameters. In the first model structure, we aggregated the parameters reflecting the age groups
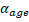
, and in the second we did not account for differences between countries, (i.e.
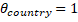
). We assessed the balance between including additional parameters and the danger of overfitting the model using the Akaike information criterion (AIC), a measure of model fitting derived from information theory[25].

We also conducted a probabilistic global uncertainty analysis to integrate the uncertainty in epidemiological parameters. Specifically, we considered epidemiological variations resulting from 1) uncertainty regarding background immunity arising from exposure in previous seasons (i.e. the initial proportion of susceptible individuals), and 2) uncertainty regarding the probability of infection given contact between susceptible and infectious persons. This was obtained by sampling the first 1000 sets of background immunity (table S2), and updating the subgroup-specific immunity,
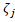
. We then independently sampled 1000 sets of annual incidence rates spanning 5-10% for adults and 20-30% for children, which is consistent with WHO estimates. For each realization, we calibrated the five parameters in the same manner described above for the base case. The marginal distribution of the calibrated parameters is presented in Figure S2.

| **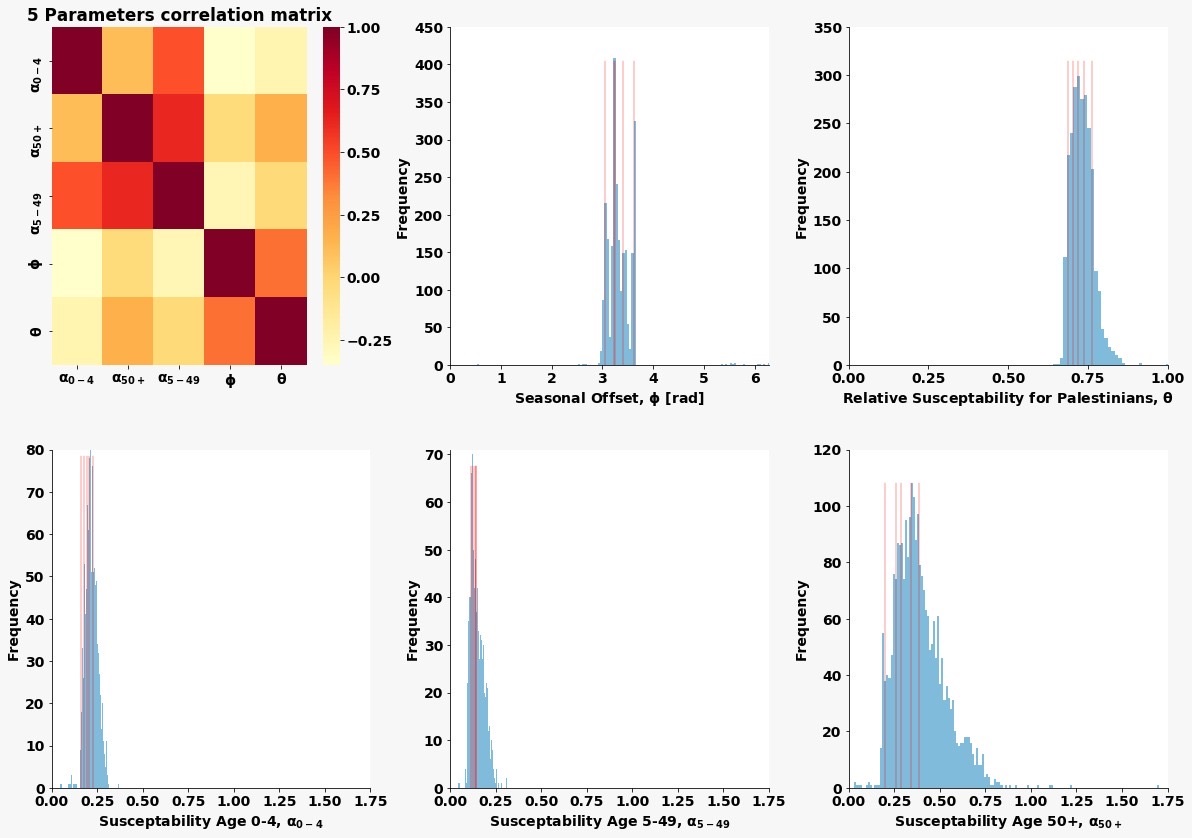** |
| --- |
| **Figure S2:** Marginal distributions of calibrated parameters, obtained using Nelder-Mead simplex algorithm. The correlation heat map emphasizes that the parameters are correlated. Thus, in each iteration of our probabilistic sensitivity analysis, parameters were jointly sampled from the five-dimensional distribution estimated. Red vertical solid lines represent parameters estimated for our base case. |

## **5. Cost-effectiveness Analysis**

The epidemiological results generated by the dynamic model simulations were integrated into an economic evaluation to estimate the cost-effectiveness of Israel subsidizing the vaccination of the Palestinian Bridge population against influenza. All the health outcome probabilities, costs and QALY losses are detailed in the table below.

Table S3: Estimated base case probabilities for health outcomes, costs and QALY losses


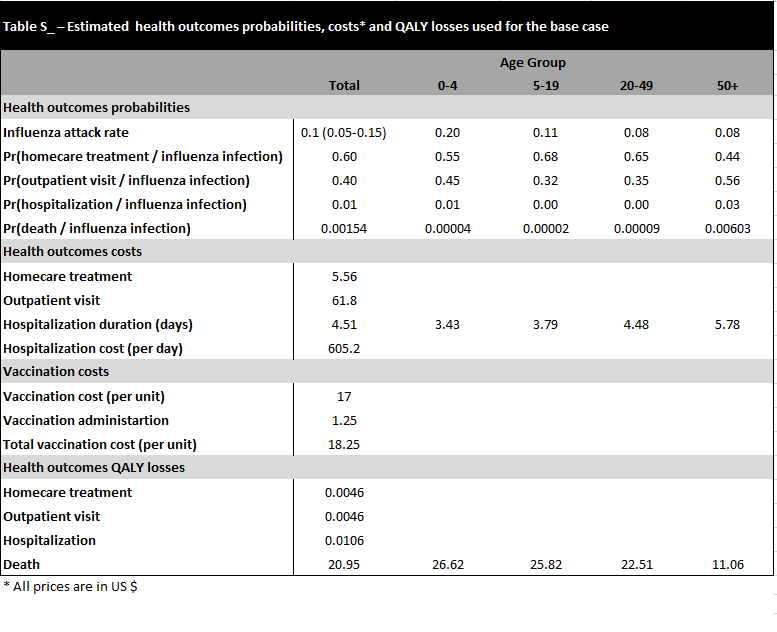


# **6. Additional results**

| To explore the effect of age on transmission, we stratified the population into four age groups (0–4 y, 5–19 y, 20–49 y, and ≥50 y) and performed a similar analysis to evaluate the mean number of secondary cases. We found that the younger age groups, particularly children under 4 years of age, were the groups in which a single case would typically transmit the disease to more than one individual (<4 y: 1.24–1.38, 5–19 y: 1.21–1.26, 20–49 y: 0.79–0.89, ≥50y: 0.47–0.54), indicating that children are predominantly responsible for transmission. In contrast, the infected elderly will transmit less than an individual in any other age group. This finding was robust across different attack rates for Israeli and Palestinians. For example, for the basecase scenario, an infected individual younger than 4 years of age is predicted to transmit influenza to 2.5 as many people as an individual older than 50 years of age (1.29 vs. 0.51, Figure S3). In addition, arising from the high-contact mixing of people within the same age group, all age groups were most likely to infect individuals in their own age group. | 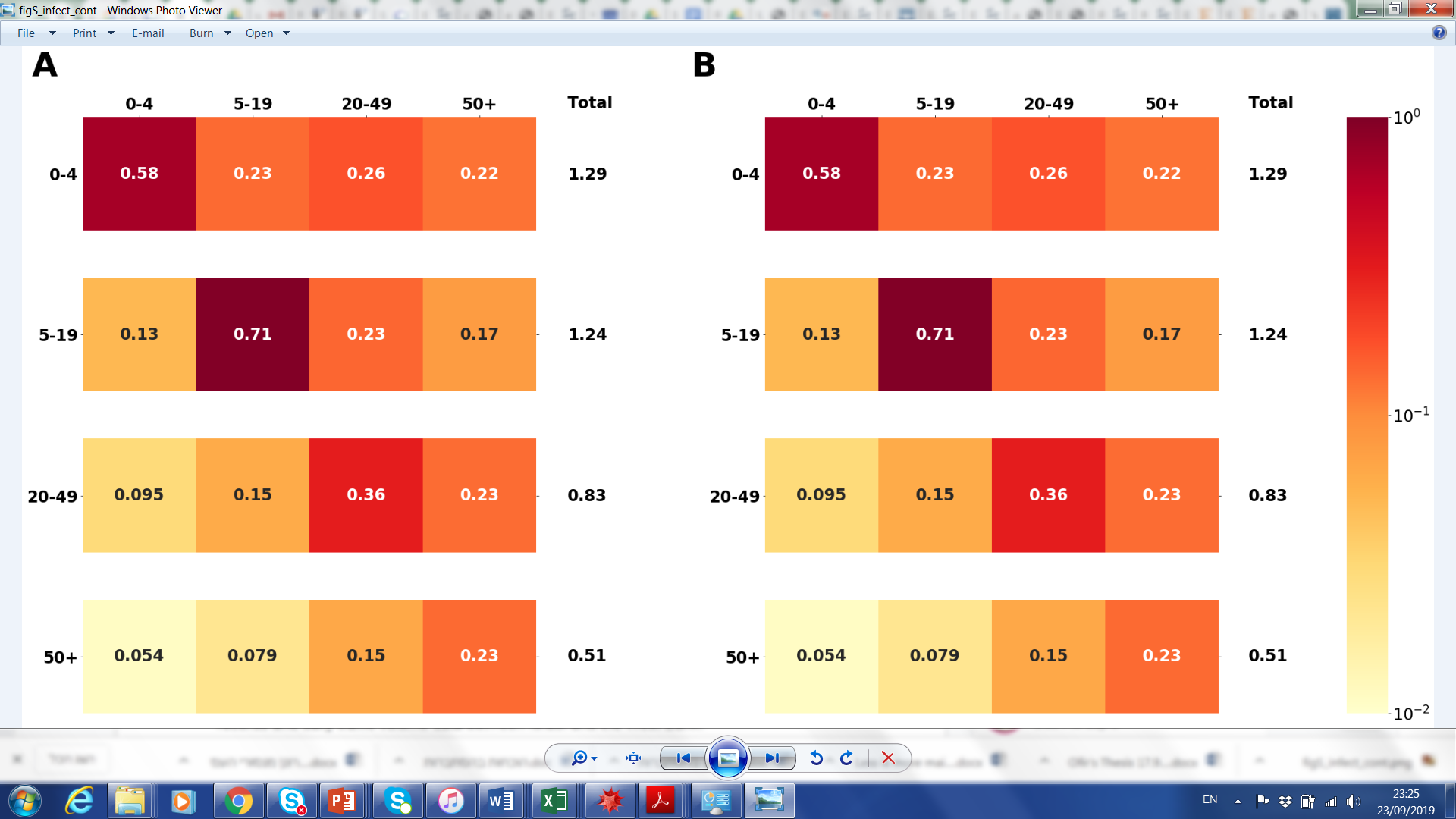 |
| --- | --- |
|  | **Figure S3.** Average number of secondary infections generated per case for each group. A row indicates the source of infection, and a column indicates the group becoming infected. The color and number at the intersection of the row and column indicate the average number of secondary infections expected from a single infective case. |

**References**

1. Seasonal Influenza Vaccine Effectiveness, 2005-2015 | Health Professionals | Seasonal Influenza (Flu).

2. Hancock K, Veguilla V, Lu X, Zhong W, Butler EN, Sun H, et al. Cross-Reactive Antibody Responses to the 2009 Pandemic H1N1 Influenza Virus. N Engl J Med. 2009;361:1945–52. doi:10.1056/NEJMoa0906453.

3. Bock Axelsen J, Yaari R, Grenfell BT, Stone L. Multiannual forecasting of seasonal influenza dynamics reveals climatic and evolutionary drivers. doi:10.1073/pnas.1321656111.

4. ראשי - מכון דו-עת - אסטרטגיה ומחקר חברתי כלכלי.

5. Mossong JL, Hens N, Jit M, Beutels P, Auranen K, Mikolajczyk R, et al. Social contacts and mixing patterns relevant to the spread of infectious diseases. PLoS Med. 2008;5. doi:10.1371/journal.pmed.0050074.

6. Ibuka Y, Ohkusa Y, Sugawara T, Chapman GB, Yamin D, Atkins KE, et al. Social contacts, vaccination decisions and influenza in Japan. J Epidemiol Community Health. 2016;70:162–7. doi:10.1136/jech-2015-205777.

7. Prem K, Cook AR, Jit M. Projecting social contact matrices in 152 countries using contact surveys and demographic data. PLoS Comput Biol. 2017;13:e1005697. doi:10.1371/journal.pcbi.1005697.

8. Kahana D, Yamin D. Accounting for the Spread of Vaccination Behavior to Optimize Influenza Vaccination Programs. SSRN Electron J. 2019.

9. Medlock J, Galvani AP. Optimizing influenza vaccine distribution. Science (80- ). 2009.

10. Ip DKM, Lau LLH, Leung NHL, Fang VJ, Chan K-H, Chu DKW, et al. Viral shedding and transmission potential of asymptomatic and pauci-symptomatic influenza virus infections in the community. Clin Infect Dis. 2016;:ciw841.

11. Lessler J, Reich NG, Brookmeyer R, Perl TM, Nelson KE, Cummings DA. Incubation periods of acute respiratory viral infections: a systematic review. The Lancet Infectious Diseases. 2009;9:291–300.

12. Furuya-Kanamori L, Cox M, Milinovich GJ, Soares Magalhaes RJ, Mackay IM, Yakob L. Heterogeneous and dynamic prevalence of asymptomatic influenza virus infections. Emerg Infect Dis. 2016;22:1052–6.

13. Leung NHL, Xu C, Ip DKM, Cowling BJ. Review Article. Epidemiology. 2015;26:862–72.

14. Lau LLH, Cowling BJ, Fang VJ, Chan K, Lau EHY, Lipsitch M, et al. Viral Shedding and Clinical Illness in Naturally Acquired Influenza Virus Infections. J Infect Dis. 2010;201:1509–16.

15. Fielding JE, Kelly HA, Mercer GN, Glass K. Systematic review of influenza A(H1N1)pdm09 virus shedding: duration is affected by severity, but not age. Influenza Other Respi Viruses. 2014;8:142–50. doi:10.1111/irv.12216.

16. Ng S, Lopez R, Kuan G, Gresh L, Balmaseda A, Harris E, et al. The Timeline of Influenza Virus Shedding in Children and Adults in a Household Transmission Study of Influenza in Managua, Nicaragua. Pediatr Infect Dis J. 2016;35:583–6.

17. CDC Seasonal Flu Vaccine Effectiveness Studies | CDC. https://www.cdc.gov/flu/vaccines-work/effectiveness-studies.htm. Accessed 3 Jan 2020.

18. Sharabi S, Drori Y, Micheli M, Friedman N, Orzitzer S, Bassal R, et al. Epidemiological and virological characterization of influenza B virus infections. PLoS One. 2016;11.

19. Antibodies Cross-Reactive to Influenza A (H3N2) Variant Virus and Impact of 2010–11 Seasonal Influenza Vaccine on Cross-Reactive Antibodies — United States. https://www.cdc.gov/mmwr/preview/mmwrhtml/mm6114a1.htm. Accessed 29 Jan 2020.

20. Mandelboim M, Bromberg M, Sherbany H, Zucker I, Yaary K, Bassal R, et al. Significant cross reactive antibodies to influenza virus in adults and children during a period of marked antigenic drift. 2014. doi:10.1186/1471-2334-14-346.

21. Blümel B, Schweiger B, Dehnert M, Buda S, Reuss A, Czogiel I, et al. Age-related prevalence of cross-reactive antibodies against influenza a(H3N2) variant virus, Germany, 2003 to 2010. Eurosurveillance. 2015;20:21206. doi:10.2807/1560-7917.es2015.20.32.21206.

22. WHO/Europe | Influenza - Data and statistics.

23. Somes MP, Turner RM, Dwyer LJ, Newall AT. Estimating the annual attack rate of seasonal influenza among unvaccinated individuals: A systematic review and meta-analysis. Vaccine. 2018;36:3199–207.

24. Gao F, Han L. Implementing the Nelder-Mead simplex algorithm with adaptive parameters. Comput Optim Appl. 2012;51:259–77.

25. Burnham KP, Anderson DR, Burnham KP. Model selection and multimodel inference : a practical information-theoretic approach. Springer; 2002.
